# The association between age and vital signs documentation of trauma patients in prehospital settings: analysis of a nationwide database in Japan

**DOI:** 10.1186/s12873-022-00725-2

**Published:** 2022-10-04

**Authors:** Mafumi Shinohara, Takashi Muguruma, Chiaki Toida, Masayasu Gakumazawa, Takeru Abe, Ichiro Takeuchi

**Affiliations:** Advanced Critical Care and Emergency Center, |Yokohama City University Medical Center, 4-57 Urafunecho. Minamiku, Yokohama City, Kanagawa 232-0024 Japan

**Keywords:** Emergency medical service, Wounds and injuries, Pediatric trauma, Prehospital care

## Abstract

**Background:**

Emergency medical service (EMS) providers are the first medical professionals to make contact with patients in an emergency. However, the frequency of care by EMS providers for severely injured children is limited. Vital signs are important factors in assessing critically ill or injured patients in the prehospital setting. However, it has been reported that documentation of pediatric vital signs is sometimes omitted, and little is known regarding the performance rate of vital sign documentation by EMS providers in Japan. Using a nationwide data base in Japan, this study aimed to evaluate the relationship between patients’ age and the documentation of vital signs in prehospital settings.

**Methods:**

This study was a secondary data analysis of the Japan Trauma Data Bank. The inclusion criterion was patients with severe trauma, as defined by an Injury Severity Score ≥ 16. Our primary outcome was the rate of recording all four basic vital signs, namely blood pressure, heart rate, respiratory rate, and level of consciousness in the prehospital setting among different age groups. We also compared the prehospital vital sign completion rate, that is, the rate at which all four vital signs were recorded in a prehospital setting based on age groups. Multivariate analysis was performed to evaluate factors associated with the prehospital vital sign completion rate.

**Results:**

We analyzed 75,777 severely injured patients. Adults accounted for 94% (71400) of these severely injured patients, whereas only 6% of patients were children. The rate of prehospital recording of vital signs was lower in children ≤5 years than in adult patients for all four vital signs. When the adult group was used as a reference, the adjusted odds ratios of vital sign completion rate in infants (0 years), younger children (1–5 years), older children (6–11 years), and teenagers (12–17 years) were 0.09, 0.30, 0.78, and 0.87, respectively.

**Conclusions:**

Analysis of the nationwide trauma registry showed that younger children tended to have a lower rate of vital sign documentation in prehospital settings.

## Background

Injury is the leading cause of death in children worldwide [1, 2]. Emergency medical service (EMS) providers are often the first medical point of contact for a patient suffering an emergency in the prehospital setting. Improving the quality of prehospital care is essential for improving patient outcomes [3, 4]. However, the frequency of care by EMS providers for severely injured children is limited. According to the Japanese annual ambulance report of 2019, the transportation of children (aged <18 years) was 8.3% (496496) of all EMS transport cases (5,978,008 cases) [5]. Therefore, EMS providers rarely treat severely injured children.

It was reported that EMS providers have heightened anxiety about the transportation of severely injured or ill children [6]. In addition, Japanese ambulances are not sufficiently equipped with medical equipment for children [7–9]. In prehospital settings, a prompt assessment of patients according to their clinical presentation rather than a focus on vital signs is important. However, vital signs are used as one of the factors of a field triage tool for critically ill or injured children in prehospital settings [10, 11]. The documentation of vital signs for pediatric patients was sometimes omitted in prehospital settings [11–15], and the rate of documentation of blood pressure (BP) was less than 50% in children aged <3 years [11, 13]. Especially in severe trauma, vital signs may play an important role in determining prehospital treatments and transport to hospitals. Even though its importance is widely acknowledged, little is known regarding the rate of vital sign documentation by EMS providers in Japan.

Thus, this study aimed to evaluate the relationship between patients’ age and the documentation of vital signs in severe trauma patients, using a nationwide database.

## Methods

### Settings

In Japan, EMS is part of the fire department and is managed by the fire defense headquarters of each local government. The Fire and Disaster Management Agency (FDMA) supervises the fire defense headquarters countrywide. To become an EMS provider, it is necessary to pass the employment examination of the local government that manages the fire defense headquarters and finish the basic curriculum for EMS providers. The learning contents for each newly hired EMS provider are decided by each fire defense headquarters and the curriculum takes about 6 months in most cases. EMS providers can assess patients with trauma and provide basic care, including chest compression, administration of oxygen, ensuring airway patency, backboard fixation, neck fixation with a neck collar, and astriction. Only emergency life-saving technicians (ELSTs) are allowed to perform specialized medical care, including intubation and insertion of intravenous lines. In Japan, ELST was established as a national qualification in 1991. To be an ELST entails graduating after two years at a vocational school or working as an EMS provider for >5 years/2000 hours, completing a 6-month course, and passing the national examination. In Japan, usually, three staff work in an ambulance as one team. It is the policy of the FDMA that, as much as possible, at least one of the three staff is an ELST. There is a medical control system for each region (in many cases it is divided municipally or by prefecture). The concrete policies of prehospital activity of EMS providers (for example how to define a patient with shock, the procedure of reporting to doctors when taking an intravenous line, etc) and the selection of hospitals in the area are discussed beforehand, according to regional medical situations by the regional medical control associations constructed by emergency medical physicians and local government and dispatch center personnel. To standardize prehospital trauma care, the Japanese Association for the Surgery of Trauma (JAST) began the Japan Prehospital Trauma Evaluation and Care (JPTEC) training course for EMS providers in 2002. The basic concept of JPTEC is to standardize prehospital trauma care and reduce preventable trauma deaths. In the course of JPTEC, physiological assessment is emphasized, and the assessment of vital signs is positioned as an important factor for the evaluation of a patient’s condition [16].

The Japan Trauma Data Bank (JTDB) is a nationwide trauma registry that was established by the Japanese Association for the JAST and the Japanese Association of Acute Medicine in 2003 aimed at improving the quality of trauma care [17]. The JTDB is managed by Japan Trauma Care and Research (JTCR) supervised by the JAST. The staff of the participating facility register cases with the JTDB online. In Japan, patient care reports by EMS providers are usually paper-based. The prehospital data are also registered by the hospital staff from paper-based records. Prehospital data include any data collected between the time the patient was loaded into the ambulance and the time the patient arrived at the hospital. The JTCR checks the validation of the data and distributes the cleansing data to the participating facilities. At the time of starting, the number of participating facilities was approximately 50, and in the 2021 JTDB report, the number of facilities submitting data to the JTDB was 295 between 2019 and 2020 [18, 19].

### Study design and variables

This study was a secondary data analysis of the JTDB. The study period was from January 1, 2009, to December 31, 2018. The inclusion criterion was patients with severe trauma as defined by an Injury Severity Score (ISS) ≥16. Exclusion criteria were as follows: patients with burns; patients with no ISS coding or age records; patients who visited a hospital with families or by themselves; patients transported by doctors’ cars (ambulances or other vehicles that are dispatched from hospitals with doctors), helicopters, or interfacility transportation; and patients with out-of-hospital cardiac arrest either at the scene or on arrival at the hospital.

The following information was obtained from the JTDB database: age, sex, year of the occurrence, ISS, Revised Trauma Score (RTS), probability of survival calculated by the Trauma Injury Severity Score (TRISS) methods (PS), presence of ELSTs during transport, and records of prehospital vital signs. The RTS and probability of survival were calculated using the vital sign readings at the time of hospital arrival retrieved from the JTDB data.

We were not able to collect prehospital oxygen saturation (SpO_2_) and temperature because these vital signs were not included in the dataset. In the JTDB database, the Japan Coma Scale (JCS) is recorded as a consciousness scale in a prehospital setting. The JCS score is a standard field tool used by EMS providers in Japan to assess patients’ consciousness, and like the Glasgow Coma Scale, is reported as a predictor of trauma patients [20, 21].

Our primary outcome was the rate of recording all four basic vital signs, namely BP, heart rate, respiratory rate, and level of consciousness in prehospital settings among different age groups. We also compared the prehospital vital sign completion rate, that is, the rate at which all four vital signs were recorded in a prehospital setting, based on age group. The age groups were categorized as follows: infants (0 years), younger children (1–5 years), older children (6–11 years), teenagers (12–17 years), and adults (≥18 years).

### Ethics approval

This study was performed in accordance with the Declaration of Helsinki. This study was approved by an Institutional Review Board, the Ethics Committee of the Yokohama City University Medical Center (B170900003). The requirement of informed consent from the patients was waived by the Ethics Committee of the Yokohama City University Medical Center /IRB because of the observational study design. In addition, all data were collected anonymously.

### Statistical analyses

Quantitative variables were expressed as median (inter quartile range: IQR). We compared all variables based on the five agegroups. We appropriately used analysis of variance, ANOVA, or the Kruskal Wallis test for continuous variables and the chi-squared test or Fisher’s exact test for categorical variables. We used the Cochran–Armitage test to analyze trends over time in the completeness of the recording of vital signs. For a significant difference, we performed multiple comparisons using the adult group as a reference. We included significant variables from the age group comparisons in the multivariable analysis. PS was used as a severity factor for avoiding multicollinearity. Thus, a multivariable logistic regression model comprised of sex, age group, year of occurrence, PS, and presence of ELSTs during transport. We checked multicollinearity of the variables by using the variance inflation factor. Statistical significance was set at *p* < 0.05. All statistical analyses were performed using STATA software (Stata/SE 13.0, StataCorp LLC, TX, USA).

## Results

**Fig. 1:**
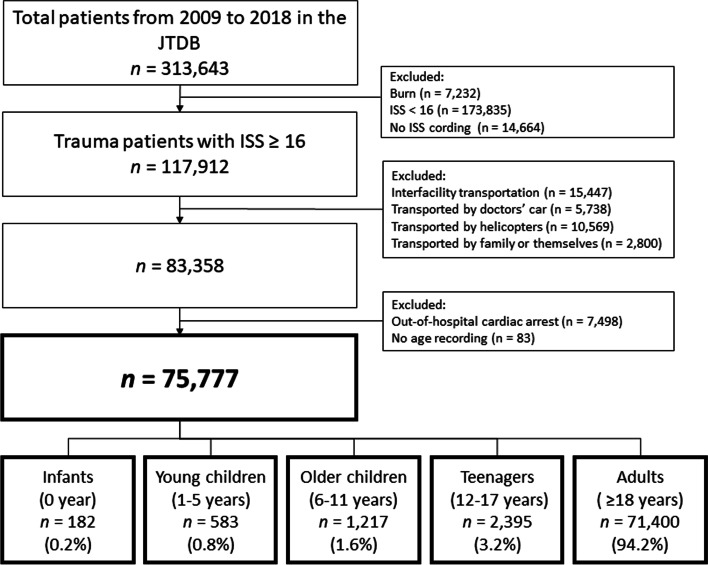
Flow-diagram of patient selection. JTDB: Japan Trauma Data Bank. ISS: Injury Severity Score

Between January 2009 and December 2018, 313,643 cases were registered in the JTDB. A total of 117,912 injured patients with an ISS ≥16, were identified. We excluded 34,554 patients with interfacility transport, who visited a hospital with their families or by themselves, or were transported by doctors’ cars and helicopters. We also excluded 7498 patients who had out-of-hospital cardiac arrests and 83 patients with no age records. Therefore, we analyzed 75,777 patients in total, of whom 0.2% (182) were infants (0 years), 0.8% (583) were younger children (1–5 years), 1.6% (1217) were older children (6–11 years), 3.2% (2395) were teenagers (12–17 years), and 94.2% (71400) were adults (≥18 years) (Fig. 1). The number of registered patients was 4671 in 2009 and 8187 in 2018. The rate of ELSTs involved in transportation was 95.2% (3165) in 2009 and 97.3% (6482) in 2018 (*p* < 0.001).

Patient characteristics according to age group are shown in Table 1. ISS was higher in teenagers and lower in children aged ≤11 years than in adult patients. The PS was higher in children than in adults. The rate of recording vital signs in a prehospital setting was lower in children ≤5 years than in adult patients for all four vital signs. In children aged ≤5 years, the vital sign completion rate was lower than that in adult patients.

**Table 1:** Patients’ characteristics and rate of documentation of vital signs by age groups

	Infants (0 year) *n* = 182	Young children (1–5 years) *n* = 583	Older Children (6–11 years) *n* = 1217	Teenagers (12–17 years) *n* = 2395	Adults (18- years) *n* = 71,400	*p* value
Sex male, n (%)	137 (75%)	369 (63%)*	832 (68%)	1737 (73%)*	49,793 (70%)	< 0.001
Years, n (%)						N/A
2009	8 (4%)	47 (8%)	92 (8%)	189 (8%)	4335 (6%)	
2010	13 (7%)	54 (9%)	115 (9%)	223 (9%)	5042 (7%)	
2011	17 (9%)	53 (9%)	117 (10%)	249 (10%)	6277 (9%)	
2012	18 (10%)	72 (12%)	150 (12%)	250 (10%)	7260 (10%)	
2013	16 (9%)	76 (13%)	163 (13%)	302 (13%)	8245 (12%)	
2014	23 (13%)	66 (11%)	129 (11%)	280 (12%)	8605 (12%)	
2015	26 (14%)	61 (10%)	131 (11%)	275 (11%)	8933 (13%)	
2016	14 (8%)	62 (11%)	119 (10%)	215 (9%)	7109 (10%)	
2017	20 (11%)	47 (8%)	115 (9%)	221 (9%)	7756 (11%)	
2018	27 (15%)	45 (8%)	86 (7%)	191 (8%)	7838 (11%)	
ISS, median (IQR)	16 (16–21) *	18 (16–25) *	20 (17–26) *	22 (17–29) *	21 (17–26)	< 0.001
Revised Trauma Score median (IQR)	6.82 (5.68–7.55) *	6.90 (5.97–7.84) *	7.55 (6.61–7.84) *	7.84 (6.32–7.84) *	7.84 (6.82–7.84)	< 0.001
Probability of survival ^a^ median (IQR)	0.97 (0.94–0.99) *	0.97 (0.91–0.99) *	0.98 (0.94–0.99) *	0.97 (0.91–0.99) *	0.92 (0.79–0.95)	< 0.001
Presence of ELSTs, n (%)	125 (92%) *	422 (94%) *	925 (97%)	1813 (97%)	53,659 (97%)	0.001
Hospital mortality, n (%)	10 (6%) *	30 (5%) *	50 (4%) *	174 (8%) *	9765 (14%)	< 0.001
Documentation of vital signs						
Blood pressure	50 (27%) *	362 (62%) *	1014 (83%)	2013 (83%)	60,291 (84%)	< 0.001
Pulse rate	133 (73%) *	481 (83%) *	1060 (87%)	2117 (88%)	62,977 (88%)	< 0.001
Respiratory rate	114 (63%) *	426 (73%) *	993 (82%)	1995 (83%)	59,463 (83%)	< 0.001
Consciousness	123 (68%) *	465 (80%) *	1077 (88%)	2142 (89%)	63,639 (89%)	< 0.001
Completion of vital signs	39 (21%) *	298 (51%) *	891 (73%)	1797 (75%)	54,076 (76%)	< 0.001

*ISS* Injury Severity Score, *IQR* Inter quartile range, *ELSTs* Emergency life saving technicians

* corrected *p* < 0.05 in multiple comparison with adults as a reference group

^a^ Probability of survival caluculated by TRISS (trauma injury severity score)

**Fig. 2:**
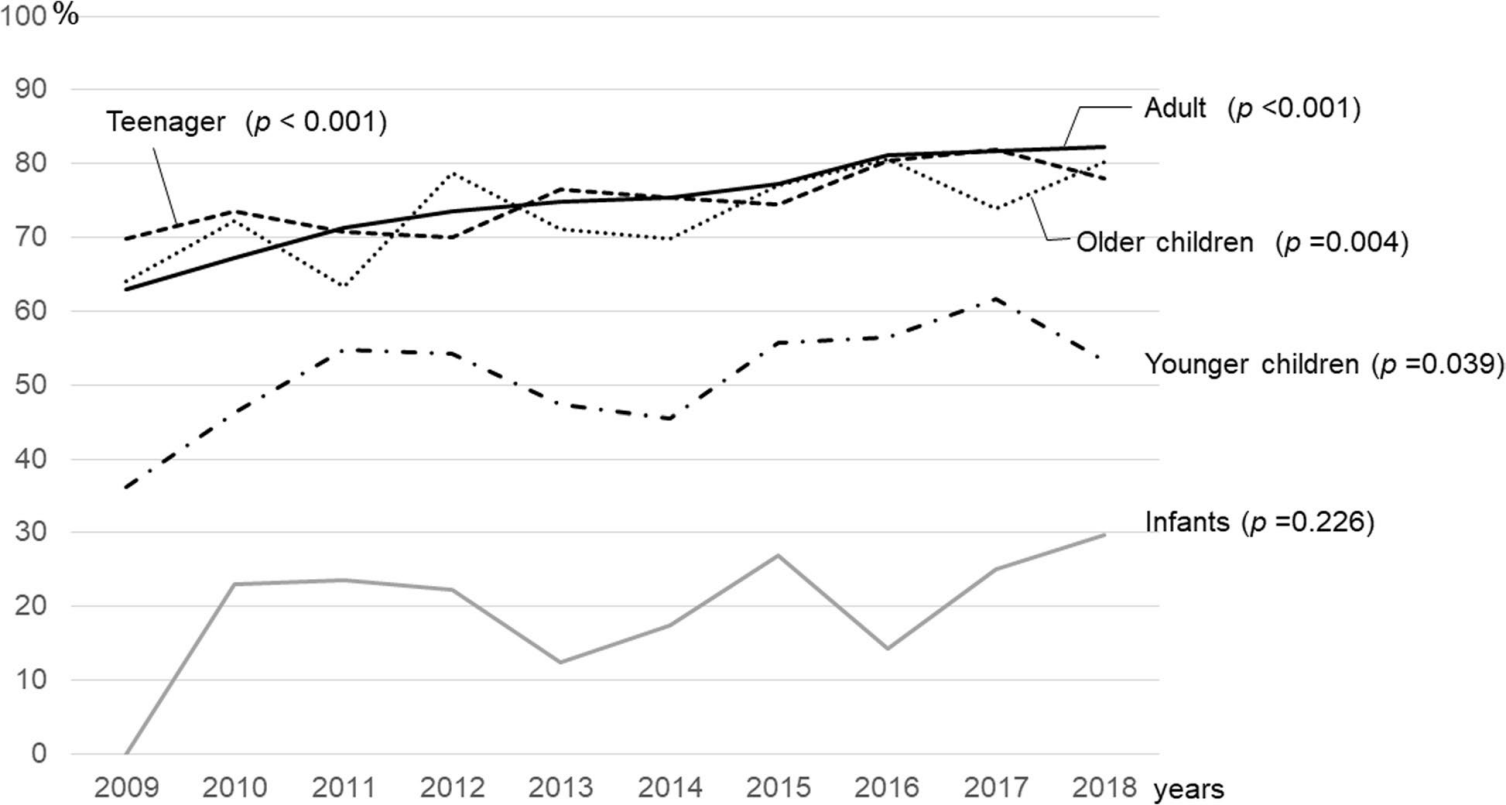
The prehospital vital sign completion rate by age group from 2009 to 2018. Chi-square’s test

The 10-year trend of the prehospital vital sign completion rate by age group is shown in Fig. 2. The completion rate of vital sign recording increased over time in adults, teenagers, older children and young children. However, in infants there was no improvement in the vital sign completion rate over the 10-year period.

Table 2 shows multivariate analysis used to evaluate the factors associated with the completion rate of vital signs in a prehospital setting. When the adult group was used as a reference, the adjusted odds ratios (ORs) of vital sign completion in infants, younger children, older children and teenagers were 0.09, 0.30, 0.78 and 0.87, respectively. Compared with 2009, there was a higher completion rate of vital signs from 2013–2018. In addition, a higher PS was associated with the completion of vital signs documentation (OR: 95% confidence interval [CI], 3.14: 2.87–3.43). The presence of ELSTs was associated with documentation of vital signs (OR: 95% CI, 1.90: 1.70–2.12). We assessed multicollinearity of the variables by using the variance inflation factor; no multicollinearity was observed.

**Table 2:** Factors associated with completion of vital signs documentation

	Odds ratio	[95% Confidence interval]	*p value*
Male	0.99	0.95–1.05	0.825
Age groups			
Infant	0.09	0.06–0.15	< 0.001
Young children	0.30	0.24–0.38	< 0.001
Older children	0.78	0.66–0.94	0.007
Teenagers	0.87	0.77–0.996	0.044
Adults	Reference		
Year			
2009	Reference		
2010	0.93	0.83–1.05	0.256
2011	1.04	0.93–1.17	0.459
2012	1.17	1.05–1.31	0.007
2013	1.34	1.20–1.50	< 0.001
2014	1.26	1.13–1.41	< 0.001
2015	1.43	1.29–1.60	< 0.001
2016	1.92	1.71–2.16	< 0.001
2017	1.84	1.64–2.06	< 0.001
2018	1.90	1.69–2.13	< 0.001
Probability of survival calculated by TRISS	3.14	2.87–3.43	< 0.001
Presence of ELSTs	1.90	1.70–2.12	< 0.001

*ELSTs* Emergency life-saving technicians, *TRISS* Trauma injury severity score

## Discussion

Our analysis of a nationwide trauma registry showed that younger children were at a higher risk of lacking vital signs documentation during prehospital trauma care than adults. Although severity, onset years, and presence of ELSTs were adjusted, the documentation of vital signs in children aged ≤11 years was less likely. In addition, the high value of PS and the presence of ELSTs were associated with the completion rate of vital signs. EMS providers rarely deal with severely injured children. This could be attributed to a low proportion of children among severely injured patients. In this study as well, pediatric patients accounted for only 6% (4377) of all patients.

Although it is imperative to promptly assess patients’ conditions according to their clinical presentation in prehospital settings, the detection of abnormal vital signs are still important. Zebreck et al. [3] reported that early (prehospital and emergency department) interventions for hypotension and hypoxia improved the outcomes of patients with moderate to severe head trauma. Vital signs are used as factors of a field triage tool for critically ill or injured children in prehospital settings [11, 16].

There are some possible explanations for the incomplete documentation of pediatric vital signs in the prehospital settings. First, it is often difficult to communicate with young children who cannot speak yet and it may create a barrier to taking vital signs [22]. Treating children in a prehospital setting requires skills and a knowledge of the behavioral characteristics of children [6, 10]. Measuring the vital signs of children requires dedicated equipment for children and pediatric-size devices are required to measure blood pressure and oxygen saturation in infants and children. A survey of the fire department in Japan revealed that medical equipment for children were insufficient in Japanese ambulances [7–9]. Second, the frequency of care by EMS providers for severely injured children is limited [5, 10]. A knowledge of pediatric physiology is required to interpret children’s vital signs and ELSTs will have studied features about children in their course [23]. However, EMS providers cannot gain sufficient experience in pediatric trauma care in on-the-job training. The vital signs of children are different from those of adults [24]. Hewes et al. reported that education for EMS providers improved the ratio of complete measurement of vital signs [15]. Educating continuously for EMS providers may promote to higher rate of measuring children’s vital signs in prehospital settings.

Our reports also showed that the vital sign completion rate has increased in the last 10 years. In addition, the presence of ELSTs during transport is associated with the vital sign completion rate. In the past 10 years, the percentage of ELSTs involved in transportation has increased. The rate of ELSTs involved in transportation was 95.2% (3165) in 2009 and 97.3% (6482) in 2018 (*p* < 0.001). A higher proportion of ELSTs involved in transportation may contribute to vital sign completion rates. In addition, the spread of education on prehospital activity represented by the JPTEC course may have promoted recognition of the importance of vital signs. EMS transport has been reported to benefit the transport of moderate to severely injured children, because of professional field triage to reach the appropriate trauma facility [4]. In Japan, trained ELSTs are also necessary for assessing injured children. However, in infants, there was no improvement in the vital sign completion rate over 10 years. The survey in 2019 showed 86% (517) of 599 fire defense headquarters had pulse oximeter sensors for a child. In addition, only 6% (38) and 55% (328) of 599 fire defense headquarters had blood pressure cuffs for neonates and infant/preschool children, respectively [9]. Distributing medical equipment, especially blood pressure cuffs for infant and young children, may lead to improving the rate of vital sign measurement in different age groups.

This study also showed that a higher PS was associated with the completion rate of vital signs documentation. In other words, documentation of vital signs tended to be omitted in more severely injured patients; this was true even after the exclusion of out-of-hospital cardiac arrest patients from our analysis. A possible rationale was that when patients were severely injured, EMS providers might have prioritized transport and omitted other vital sign measurements.

Our study had several limitations. First, even though the database is nationwide-based, it cannot cover all injured and transported patients. The number of JTDB registrations is increasing annually, however, trauma patients transported by ambulance and trauma deaths have not increased significantly in the last 10 years [2, 5]. It has been suggested that older data might not reflect the trauma care at that time. In addition, many of the facilities participating in the JTDB have trauma specialists. On the contrary, rural or small-scale facilities that do not have a trauma specialist might not be included in the JTDB. From the report of the FDMA, transport time varies by area, and in more urban areas transport takes longer. Longer transport times might promote the acquiring of vital signs; This could have caused selection bias and underestimated the prehospital vital sign completion rate. Second, the reason for the missing vital signs in the database is unknown. When there are no vital signs data in the database, it is possible that either the EMS providers did not measure the vital signs or the hospital staff who registered the data neglected to register them in the database. In addition, the JTDB data set did not include prehospital SpO2 and temperature, which are important prehospital vital signs to assess the patient’s condition. Third, there were few pediatric patients with severe trauma. Due to the small sample size, the power may be insufficient to detect significant differences in the performance rate. Finally, the missing data of the JTDB might have impaired the precision of the analysis.

## Conclusion

Analysis of the nationwide trauma registry showed that younger children tended to have a lower rate of vital sign documentation in a prehospital setting. The findings of this study could contribute to identifying problems in pediatric care and developing countermeasures for injured children in a prehospital setting.

## Data Availability

The data that support the findings of this study are available from Japan Trauma Data Bank but restrictions apply to the availability of these data, which were used under license for the current study, and so are not publicly available. Data are however available from the corresponding author upon reasonable request and with permission of the Japan Trauma Data Bank.

## Abbreviations

EMS: Emergency medical service
BP: Blood pressure
FDMA: The Fire and Disaster Management Agency
ELST: Emergency life-saving technician
JAST: Japanese Association for the Surgery of Trauma
JPTEC: Japan Prehospital Trauma Evaluation and Care
JTDB: The Japan Trauma Data Bank
JTCR: Japan Trauma Care and Research
ISS: Injury Severity Score
RTS: Revised Trauma Score
TRISS: Trauma Injury Severity Score
PS: Provability of survival calculated by the Trauma Injury Severity Score
SpO_2_: Oxygen saturation
JCS: Japan Coma Scale
IQR: Inter quartile range
OR: Odds ratio
